# Neural correlates of social affect and social cognition as risk markers of bipolar disorder

**DOI:** 10.1192/bjp.2024.282

**Published:** 2026-02

**Authors:** Dahna Choi, Katharina Förster, Malin Katharina Hildebrandt, Lara Zoë Maliske, Konrad Lehmann, Philipp Kanske, Emanuel Jauk

**Affiliations:** Institute of Clinical Psychology and Psychotherapy, TUD Dresden University of Technology, Germany; Department of Psychology, Faculty of Psychology and Educational Sciences, Babeş-Bolyai University, Romania; Department of Medical Psychology, Psychosomatics, and Psychotherapy, Medical University of Graz, Austria

**Keywords:** Bipolar disorder, empathy, endophenotypes, magnetic resonance imaging, theory of mind

## Abstract

**Background:**

The identification of early warning signs is of great importance for identifying individuals at risk for mental disorders. Especially in the case of bipolar disorder, these research endeavours are imperative considering that the frequently delayed diagnoses and longer illness duration are associated with symptom exacerbation and lower recovery rates.

**Aims:**

To multimodally investigate associations between hypomanic personality traits and altered social affect and social cognition to probe their role as early warning signs of bipolar disorder.

**Method:**

In a community sample (*n* = 140; 50.71% female), we investigated associations between hypomanic personality traits and both behavioural and neural activity measures of empathy and theory of mind (ToM) based on data from a functional magnetic resonance imaging paradigm.

**Results:**

Although analyses revealed no significant associations between behavioural or neural correlates of empathy and hypomanic personality traits, these traits were significantly associated with elevated ToM-related neural activity in the anterior rostral medial prefrontal cortex and anterior cingulate cortex. These neural activation differences were not accompanied by differences in behavioural ToM performance, suggesting more intense recruitment of task-relevant brain regions but unaffected behavioural outcomes.

**Conclusions:**

Our findings indicate hypomanic personality traits to be positively associated with ToM-related neural activity but not with behavioural ToM performance. Prospectively, our study contributes to driving towards a more comprehensive and potentially neurobiologically grounded phenotype of bipolar disorder risk that contributes to a more differential understanding of risk and resilience mechanisms.

Bipolar disorder is among the leading causes of disability worldwide, entailing an immense individual and economic burden. Unfortunately, the diagnostic process is often delayed and erroneous, which results in longer illness durations and lower remission rates.^1^ A promising approach to foster more timely diagnoses is to do research on endophenotypes, which describe behavioural, affective or cognitive characteristics that are heritable, state-independent and overly represented in individuals with an elevated risk of illness.^2^

Two putative endophenotypes relevant to mental disorders are altered functioning in social affect and social cognition.^3^ In bipolar disorder, socio-cognitive deficits and interacting emotion-related impairments have been hypothesised to contribute to the development of mania and have also been shown to be present during remission.^4^ More specifically, bipolar disorder is thought to be related to elevated emotional reactivity and maladaptive emotion regulation.^4^ Especially in interaction with bipolar disorder-related cognitive deficits,^4^ such high emotionality yields a high risk of triggering affective episodes.

In our research, we focus on two core aspects of social affect and social cognition. Empathy, on the one hand, is defined as sharing another’s emotional state in a vicarious and isomorphic way.^5^ Although empathic affect sharing can result in adaptive, caring reactions of empathic concern – also termed compassion – it can lead to empathic distress, reflecting an aversive and self-oriented response that yields, when experienced chronically, negative mental health outcomes.^6^ Theory of mind (ToM), on the other hand, refers to the ability to cognitively represent and reason about other people’s mental or affective states, which is a crucial capacity for interpersonal communication and maintenance of social contact.^7^ Considering the malleability of socio-affective and socio-cognitive abilities and their relevance for social functioning, these putative endophenotypes are promising in their clinical applicability as treatment targets.

Although empathy and ToM are sometimes more broadly subsumed under the term social cognition, we opt for a clear differentiation of affective versus cognitive routes to understanding others in the light of empirical findings on their neural distinctiveness.^3^ Against the background of this specificity in underlying neural correlates, especially when studying those impairments in individuals without manifest symptomatology, neuroimaging can be expected to provide valuable insights, since subthreshold variations might be too subtle to be detectable on an experiential or behavioural level. For example, in a review on explicit emotion regulation in bipolar disorder, impairments on a neural but not on a behavioural level have been reported,^8^ rendering promising the incorporation of neuroimaging in our own study.

Neurobiological models of bipolar disorder suggest symptoms of affect lability and cognitive deficits to result from hypoactivity in brain regions associated with emotion regulation or attentional control, including the orbitofrontal or medial prefrontal cortex (mPFC) and hyperactive subcortical structures such as the amygdala or ventral striatum.^9^ Less research has been conducted in at-risk populations. Some studies indicate similarly aberrant fronto-limbic functional connectivity; however, findings on the direction and location of neural impairments are conflicting. More conclusive findings are evident regarding brain networks related to empathy and ToM, largely overlapping with the salience and default mode network. Among others, brain regions associated with empathy comprise the inferior frontal gyri (IFG) and insulae, while the cortical midline and temporoparietal areas show activation at ToM demands.^3^ Considering the lack of neuroimaging findings of empathy and ToM in populations at risk for bipolar disorder, we take these meta-analytically validated findings as a starting point for our region of interest (ROI) analyses, with our specific hypotheses further being inferred from behavioural findings.

## Empirical findings on empathy and ToM in association with bipolar disorder

Most studies on populations at risk for bipolar disorder have reported findings on behavioural and self-report measures of empathy and ToM. For example, first-degree relatives of people with bipolar disorder have shown impairments in ToM performance compared with healthy controls.^10^ This aligns with findings on ToM deficits in people with bipolar disorder^11^ that can be additionally considered since at-risk populations frequently show intermediate phenotypes between healthy controls and people with bipolar disorder.

Although to our knowledge, no studies have investigated populations at risk for bipolar disorder regarding our conceptual understanding of empathy, related findings indicate risk-related aberrant emotional downregulation and emotional reactivity, and elevated attentional interference by task-irrelevant emotional stimuli.^12,13^ Based on these findings, emotional hyperresponsiveness has been suggested as a candidate endophenotype in bipolar disorder risk, potentially indicating similarly elevated empathic responses in this risk. Among people with bipolar disorder, results on empathy are less consistent compared with those on impaired ToM, but they predominantly indicate elevated empathy and empathic distress.^14^ As for empathic concern, impaired, elevated and no significant associations with bipolar disorder have all been reported.^14,15^ Although the evidence is inconclusive, alterations in empathy and ToM are plausible mechanisms potentially fostering manic or depressive symptoms: an overall elevated empathic responding towards another’s emotions might reinforce affective lability in individuals at risk of bipolar disorder,^16^ and this affective lability might be exacerbated by poor ToM capacities required to more cognitively reflect on others’ emotions.

Importantly, indications of socio-affective and socio-cognitive impairments have been shown across different disorder states.^11,17^ Also in a recent longitudinal study, euthymic people with bipolar disorder compared with healthy controls have shown lower scores on the Reading the Mind in the Eyes Test (RMET) over a 4.5 year period, which was interpreted as a measure of affective ToM^18^ (see^3,19^ for discussion of the RMET). Neither acute symptomatology nor past episodes had a significant influence on the severity of impairments,^18^ which again indicates the stability of those alterations and their potential endophenotypic value.

To follow up on these indications, we aim to investigate such potential socio-affective and socio-cognitive alterations in bipolar disorder risk. As in previous studies, we operationalise bipolar disorder risk using hypomanic personality traits, the latter describing energetic and upbeat traits associated with higher gregariousness, ambition and self-esteem.^20^ Although not all people with bipolar disorder show hypomanic traits premorbidly, these have shown to be predictive for conversion to bipolar disorder over a 13-year period.^20,21^

## Research aim and hypotheses

In this study, behavioural and neural correlates of empathy and ToM measured using an experimental functional magnetic resonance imaging (fMRI) task (EmpaToM)^22^ were investigated in association with hypomanic personality traits. We hypothesised that individuals with higher levels of hypomanic personality traits would show increased levels of empathy and impaired ToM performance. Regarding neural associations, we hypothesised that higher versus lower behavioural scores would be reflected in increased versus decreased neural activity respectively, since positive correlations between neural activity and behavioural empathy and ToM have been demonstrated in previous investigations.^22,23^ Based on prior research findings,^3,22,24^ we predefined ROIs for the respective analyses (see preregistration: https://osf.io/kh2tv).

In an exploratory scope, self-report data on dispositional social affect and social cognition were considered in their association with hypomanic personality traits. For a preliminarily exploration of the clinical implications of hypomanic personality traits, their different dimensions were investigated in their associations with symptom load and life-time mental disorders.

## Method

### Study procedure and participants

Individuals from the general population were invited to take part in a study on ‘personality and interpersonal interaction’ (see Supplementary material; also^23,25^). The study adhered to ethical standards of the national and institutional committees on human experimentation and with the Helsinki Declaration of 1975, as revised in 2013, and was approved by the Ethics Committee of TUD Dresden University of Technology (reference no. EK133042018). Written informed consent was obtained from all participants.

Study participants were required to be aged between 18 and 60 years, right-handed and have normal or corrected to normal vision. We excluded individuals with MRI contraindications, neurological disorders or severe mental disorders and those currently taking psychotropic medication. Sociodemographic data, hypomanic personality traits, behavioural empathy and ToM results for the final study sample (*n* = 140) are depicted in Table 1.

**Table 1:** Descriptive statistics of sociodemographic characteristics, hypomanic personality traits and behavioural measures of empathy and theory of mind

Variable		Descriptive statistics		
		Mean	s.d.	Range
Sociodemographic characteristics				
Gender (f/m/div), *n*	71/69/0			
Age, years		25.35	6.56	18–57
Hypomanic personality traits				
HPS sum score		22.14	4.49	15–36
HPS social vitality		9.81	2.13	5–16
HPS mood volatility		6.96	2.43	2–14
HPS excitement		3.69	1.34	1–8
Social affect and social cognition				
Empathy		1.98	0.87	−0.07 to 4.16
Theory of mind		0.68	0.12	0.38 to 0.92

div, diverse; f, female; HPS, Hypomanic Personality Scale; m, male.

Behavioural data from the EmpaToM paradigm.

### Psychometric measures

Hypomanic personality traits were assessed with the German Hypomanic Personality Scale (HPS);^20,26^ Both the HPS sum score and scores on three subscales were considered; the subscales were Social Vitality (social potency and vivaciousness), Mood Volatility (negative, unpredictable mood states) and Excitement (energetic and extremely cheerful mood).^27^

Self-reported dispositional social affect and social cognition were assessed with the 16-item German Interpersonal Reactivity Index (IRI),^28^ which is one of the most commonly used self-report scales to measure empathy.^29^ It comprises four subscales: Empathic Concern (feeling of sympathy and concern for others), Personal Distress (self-oriented feelings of personal anxiety and unease in tense interpersonal settings), Perspective Taking (tendency to adopt the psychological point of view of others) and Fantasy (tendency to transpose oneself imaginatively into the feelings and actions of fictitious characters.^30^ In accordance with previous publications on this data-set,^23,25^ IRI empathic concern and IRI personal distress were considered as indicators of social affect, and IRI perspective taking was considered as an indicator of social cognition.

As a measure of symptom load, the Global Severity Index of the German Brief Symptom Inventory (BSI-GSI)^31^ was analysed. The presence of past or present mental disorders was assessed via one item asking study participants whether they have ever been diagnosed with a mental disorder (see Supplementary material for details on the measures’ psychometrics).

### The fMRI paradigm

**Fig. 1:**
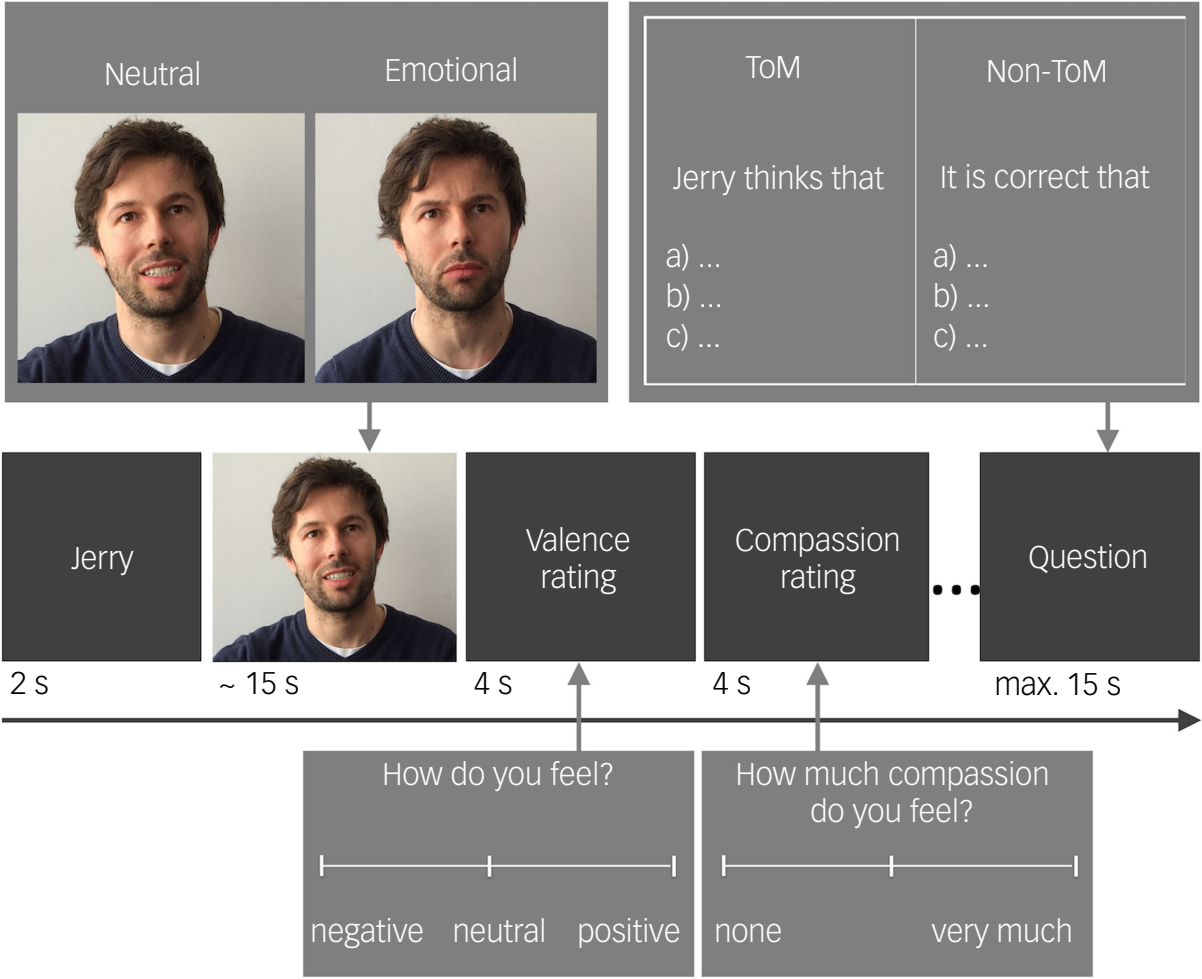
A trial sequence used in the EmpaToM paradigm. Participants are presented with a fixation cross (1–3 s), followed by the name of a narrator (1 s), who is talking about an allegedly autobiographical, emotionally negative or neutral event in a subsequently presented video (∼15 s). Participants are asked about the valence of their emotions after watching each video (visual analogue scale from negative to positive; 4 s) and about the extent of compassion they feel for the person presented in the video (visual analogue scale from none to very much; 4 s). Subsequently, another fixation cross (1–3 s) and a multiple-choice question with three response options is presented. Answering the question demands either theory of mind (ToM) or factual reasoning on the content of the previously presented video, representing the ToM and non-ToM condition respectively. More specifically, ToM-questions require inferences on true and false beliefs, preferences and desires, irony, sarcasm, metaphors, (white) lies, deception and faux pas. A maximum of 15 s is provided to indicate an answer to the questions. Thus, the paradigm follows a 2 × 2 factorial design with 12 trials per each of the four conditions, differing in the emotionality of the videos (negative versus neutral) and in what question they give rise to (ToM versus non-ToM). The duration of the task is about 30 min (adapted from^22^).

To investigate empathy and ToM, we used the EmpaToM paradigm.^22^ In this naturalistic fMRI paradigm, participants are presented with video stimuli in which a narrator talks about an allegedly autobiographical, either emotionally negative or neutral, event. Behavioural measures of empathy and ToM are derived from subsequent ratings on the participant’s emotional valence after watching the video and multiple-choice questions on the content of the video (Fig. 1).

On a behavioural level, the main outcome measures comprise behavioural empathy (i.e. the reverse-coded difference in a participant’s emotional valence ratings after emotionally negative versus neutral videos), empathic concern (i.e. compassion ratings after each video) and ToM performance (i.e. accuracy rates in the ToM conditions). Regarding the fMRI data, empathy-related neural activity is extracted by contrasting neural activity during the emotionally negative versus neutral video sequences. ToM-related neural activity is extracted by contrasting ToM versus non-ToM question sequences.

### MRI data acquisition

MRI data were acquired using a 3 T Siemens Tim Trio scanner (Siemens Healthcare GmbH, Erlangen) with a 32-channel head coil. For the structural scan, a T1-weighted sequence (repetition time 2300 ms; echo time 2.98 ms; inversion time 900 ms; flip angle 9°; 176 sagittal slices; matrix size 256 × 256; field of view (FOV) 256 mm; slice thickness 1 mm) was used. For the functional imaging in the fMRI paradigm, a T2*-weighted EPI sequence was used (repetition time 2360 ms; echo time 27 ms, flip angle 90°). Thirty-seven axial slices covering the whole brain (slice thickness 3 mm; in-plane resolution 3 × 3 mm; interslice gap 1 mm; FOV = 210 mm; matrix size 70 × 70) were acquired.

### ROI selection

We performed preregistered ROI analyses, complemented by exploratory whole-brain analyses. To ensure a systematic ROI selection and independence from the present data, previous findings on (a) task-related activity in the EmpaToM,^22^ (b) respective meta-analytic findings on empathy- and ToM-related neural activity^3^ and (c) emotion- and cognition-related neural impairments observed in populations at risk for bipolar disorder^24^ were jointly considered. We chose spherical ROIs with a fixed diameter of 7 mm rather than anatomical regions to keep the number of voxels constant across the statistical tests.

In a multi-step selection procedure (see Supplementary material), we chose the bilateral IFG pars orbitalis, bilateral angular gyri, precuneus and left cerebellum as empathy ROIs, and bilateral triangular parts of the IFG, left posterior cingulate gyrus, two areas in the middle temporal gyrus, right cerebellum and bilateral medial parts of the superior frontal cortex as while ToM ROIs (Supplementary Table S1).

### fMRI data analysis

For fMRI data analysis, we used SPM 12 for Windows (www.fil.ion.ucl.ac.uk/spm/software/spm12/). Preprocessing, the first- and second-level analyses described in the Supplementary material were conducted in accordance with our previous studies.^23,25^ Brain activation was analysed for the ‘empathy contrast’ (emotionally negative versus neutral videos) and the ’ToM contrast’ (ToM versus non-ToM questions). In the ROI analyses, activation values were extracted using the MarsBaR toolbox for SPM.^32^

### Statistical analyses and inference criteria

All further analyses were conducted using R statistical software (version 4.3.1 for Windows ). In multiple linear regressions, gender and age were added as control covariates. For the fMRI analyses, linear regressions were calculated for each ROI separately. Dependent variables in the exploratory analyses comprised the EmpaToM empathic concern rating, IRI subscale scores and the BSI-GSI. An independent samples *t*-test was conducted to test for differences on the HPS in participants with versus without a history of mental disorders. All analyses were carried out for the HPS sum score as well as the HPS subscales.

Analogous to previous work,^23^ exploratory whole-brain analyses including both cortical and subcortical structures were conducted at a voxel-level threshold of *P* < 0.001 (uncorrected) with a cluster threshold of *k* > 10 contiguous voxels. For the linear regression and *t*-test analyses, a standard threshold of *P* < 0.05 (uncorrected) was applied.

## Results

### Hypomanic personality traits, empathy and ToM

**Fig. 2:**
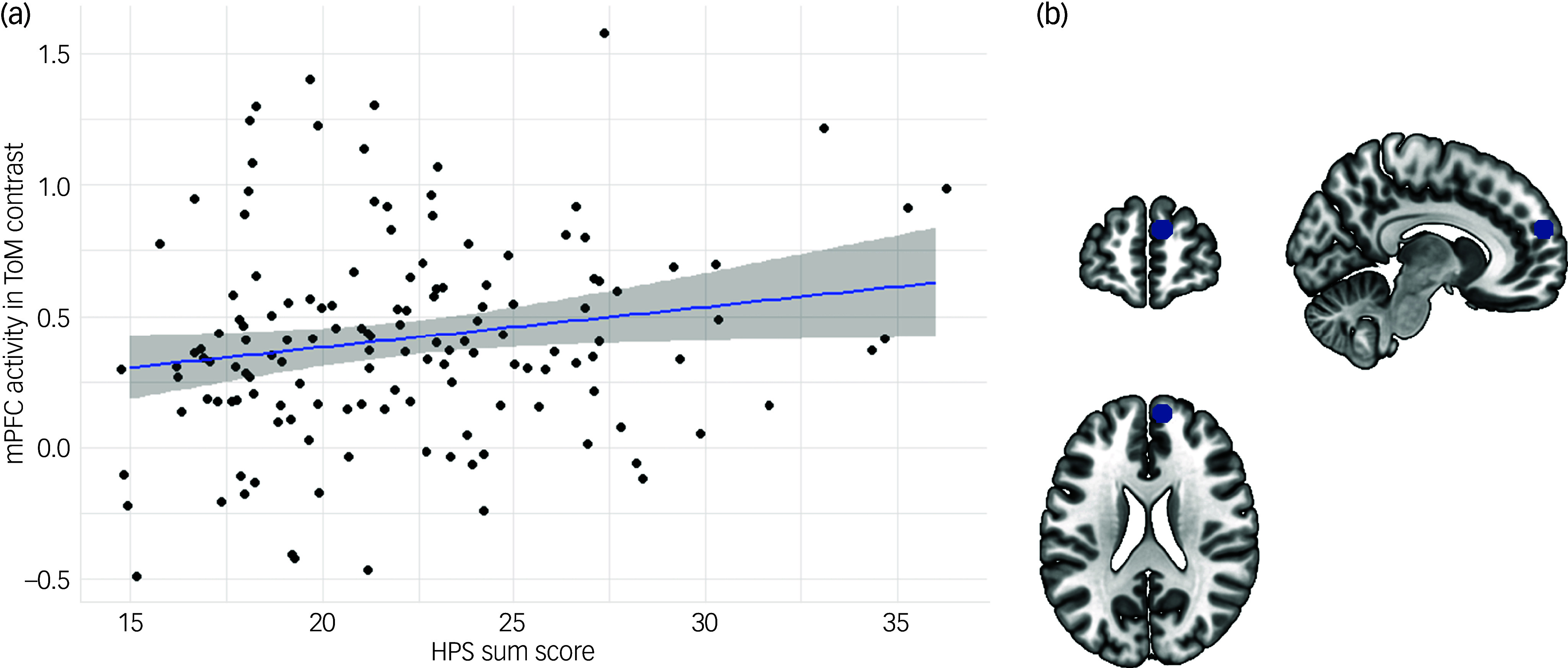
Results from linear regression analyses on hypomanic personality traits on theory of mind (ToM)-related neural activity. (a) Scatter plot depicting the association between the Hypomanic Personality Scale (HPS) sum score and neural activity in the right medial prefrontal cortex (mPFC) in the ToM contrast of the EmpaToM paradigm. (b) Schematic depiction of ToM region of interest (ROI) in the right mPFC.

**Fig. 3:**
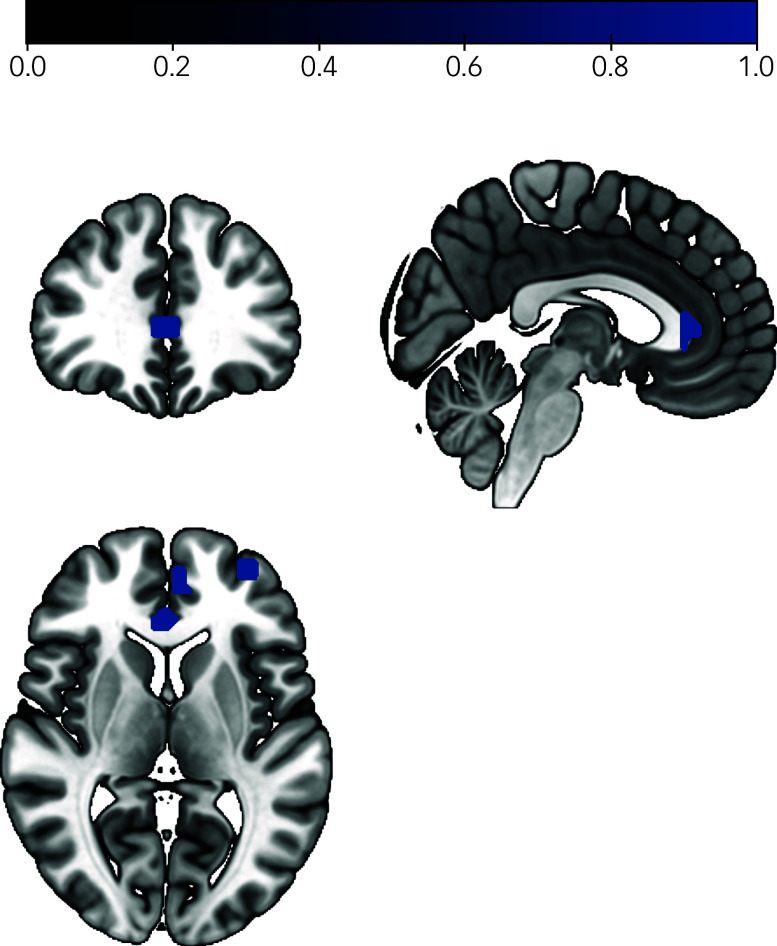
Results from whole-brain analyses on the effect of hypomanic personality traits on theory of mind (ToM)-related neural activity.

Linear regression analyses revealed a significant effect of the HPS sum score on neural activity in the ToM contrast in the right mPFC (*β* = 0.171, *P* = 0.046; Fig. 2). There were no significant effects of the HPS on neural activity in any of the other ToM ROIs (*P*_s_ = 0.166–0.948). Results from exploratory whole-brain analyses indicate further ToM-related neural activity to be positively associated with the HPS, mainly located in a cluster in the rostral anterior cingulate cortex (ACC) (*x* = 3, *y* = 33, *z* = 6; *k* = 31, voxel level: *P* < 0.001; Fig. 3 and Table S2). Analyses yielded no significant results regarding the effect of the HPS on behavioural ToM performance (*P* = 0.582).

No significant association between the HPS and neural activity in the empathy contrast was evident for any of the predefined ROIs or at a whole-brain level (*P*_s_ = 0.227–0.806). Similarly, there was a non-significant effect of the HPS on behavioural empathy (*P* = 0.487; see Tables S3–S6 for results of all ROI analyses).

### Hypomanic personality traits and self-reported dispositional social affect and social cognition

For IRI empathic concern, analyses revealed a positive effect of the HPS sum score (*β* = 0.233, *P* = 0.003). In the linear regression models in which the three subscales were considered separately, both HPS mood volatility (*β* = 0.204, *P* = 0.009) and HPS excitement (*β* = 0.222, *P* = 0.004) were positively associated with IRI empathic concern, whereas HPS social vitality alone yielded no significant effect (*P* = 0.160). There was no significant effect of any of the HPS measures on IRI perspective taking (*P*_s_ = 0.112–0.352) or IRI personal distress (*P*_s_ = 0.329–0.907).

### Hypomanic personality traits and empathic concern in the EmpaToM task

Analyses on the association between the HPS scores and ratings of empathic concern in the EmpaToM revealed no significant association in the model in which the HPS sum score was considered as a whole (*P* = 0.257). In the model in which the three HPS subscales were separately considered, HPS excitement revealed a significant positive association with empathic concern (*β* = 0.295, *P* < 0.001), but there was no significant effect of HPS social vitality (*P* = 0.993) or HPS mood volatility (*P* = 0.745).

### Hypomanic personality traits and mental health status

Results from separate multiple regression analyses on the effect of the HPS scores on the BSI-GSI demonstrated the HPS sum score (*β* = 0.327, *P* < 0.001), HPS social vitality (*β* = 0.395, *P* < 0.001) and HPS mood volatility (*β* = 0.265, *P* = 0.002) to be positively associated with the BSI-GSI, whereas HPS excitement did not yield a significant effect (*P* = 0.800). Similarly, group differences in the sense of higher hypomanic personality traits in study participants with (*n* = 27) versus without (*n* = 113) a history of mental disorders were evident for the HPS sum score (*t*(138) = −2.446, *P* = 0.016), HPS social vitality (*t*(138) = −3.216, *P* = 0.002) and HPS mood volatility (*t*(138) = 0.433, *P* = 0.016), but not HPS excitement (*P* = 0.666).

## Discussion

In this preregistered study, we explored alterations in social affect and social cognition in association with risk for bipolar disorder. To this end, behavioural and neural measures of empathy and ToM were investigated in association with hypomanic personality traits as a marker of bipolar disorder risk. For a multimodal insight, self-reported dispositional social affect and social cognition were additionally considered.

Analyses revealed hypomanic personality traits to be positively associated with ToM-related neural activity in anterior rostral parts of the right mPFC. Additionally, exploratory whole-brain analyses revealed a positive association between hypomanic personality traits and ToM-related neural activity mainly located in the rostral ACC. Behavioural ToM performance did not show significant associations with hypomanic personality traits. Regarding behavioural or neural measures of empathy, results do not support the notion of elevated empathy in hypomanic personality. However, self-reported empathic concern as another aspect of social affect was positively associated with hypomanic personality traits.

Our findings on ToM-related neural activity in the mPFC and ACC can be discussed in consideration of previous findings of these brain regions’ functional associations. The mPFC and ACC have been associated with cognitive functions related to working memory, decision-making and cognitive flexibility.^33^ As for the mPFC as a central hub in social cognition, anterior rostral compared with more posterior parts have shown activation in social cognition tasks, comprising self-knowledge, person perception and mentalising.^34^ Although the adjacent ACC is included in most reviews on the mPFC, in more specific investigations, activity in the rostral ACC has been reported to be associated with social information processing.^35^ This is supported by animal studies showing ACC lesions to lead to reduced interest in acquiring social information.^36^ Taken together, our findings correspond to previous findings on the rostral mPFC’s and ACC’s functional associations related to social cognition.

Potential implications of these findings on aberrant neural activity for bipolar disorder risk are to be evaluated in the light of no differences in behavioural measures. Conceivably, elevated frontal brain activity during ToM requirements could be indicative of higher amounts of resources required to achieve the same behavioural outcome.^36^ When evaluating potential underlying mechanisms, it is important to note that our results do not indicate the hypothesised negative but a positive association with bipolar disorder risk. In contrast to a lower ToM in people with bipolar disorder, on which we based our hypotheses, elevated activity might be indicative of an adaptive compensation of predisposition to risk, enabling unimpaired behavioural outcomes. Put differently, bipolar disorder risk-related proneness to show impaired ToM performance might be partly resolved or counteracted by higher cognitive effort, reflected in elevated neural activity.

### Indications on psychopathological value of hypomanic personality traits

In previous research, hypomanic personality has been discussed regarding its double-edged nature: on the one hand, higher levels of hypomanic personality traits entail an elevated risk for developing bipolar disorder, whereas on the other hand they are considered to be adaptive at moderate levels.^20^

Although preliminary, results from our exploratory analyses point to a similar direction, with neither the HPS sum score nor the subscales showing an association with self-reported IRI personal distress but with IRI empathic concern. As empathic concern is related to more adaptive outcomes such as prosocial tendencies and lower aggression,^37^ these findings might indicate hypomanic personality traits to be associated with adaptive aspects of empathic affect sharing. Greater gregariousness and social activity as facets of hypomanic personality might entail a higher urge to seek out social interactions and higher interest in social relationships. Conceivably, this could enable individuals at risk for bipolar disorder to show higher empathic concern without tilting into maladaptive empathic distress observed in people with bipolar disorder. As our findings are based on individuals’ self-reports, however, potential subjectivity biases are to be considered. Whether subjective reports of higher empathic concern translate into effectively higher levels in real-life social interactions requires further investigation.

Further differential insight is provided in our findings on the HPS subcomponents’ differential associations with empathic concern, which might reflect their differential psychopathological value. More specifically, the HPS sum score, HPS mood volatility and HPS excitement yielded significant associations with empathic concern, whereas HPS social vitality did not. In a similarly differential pattern, only HPS mood volatility and HPS social vitality were positively associated with symptom load and life-time psychiatric diagnoses. Although we do not claim to conclusively interpret these results on the level of HPS subscales owing to the overall moderate HPS scores in our sample, we point out the observation that the HPS subscales reveal associations that are not evident when merely considering the composite measure as a whole. We suggest investigating hypomanic personality traits in their multidimensional structure in order to explore their presumably differential relevance for disorder onset or even their protective value against it.

### Limitations and future directions

As our cross-sectional study design does not allow for conclusions regarding causal associations, longitudinal investigations on the effective functional implications of neural variations in relation to development of bipolar disorder are needed. Moreover, the overall right-skewed distribution of HPS scores in our sample impedes drawing generalised conclusions about at-risk populations. Although the mean level of hypomanic personality traits in our sample is similar to previous investigations,^20,27^ hypomanic personality traits might – in spite of their dimensional rather than categorical nature – reflect relevant bipolar disorder risk only when exceeding a certain threshold. At-risk populations should be specifically recruited and studied with more conservative statistical correction approaches to follow up on our findings’ indications. Prospectively, at-risk populations as well as healthy controls and individuals with bipolar disorder should be jointly investigated to disentangle mechanisms of predisposition, resilience and disease expression. The specificity of our findings for bipolar disorder risk versus such alterations’ potential as a transdiagnostic marker is to be investigated in contrast to risks for other mental disorders.

On a more specific note, the EmpaToM enables investigation of the sharing of negatively valenced stimuli, which precludes the generalisability to empathy for positive emotions. The possibility of valence-specific alterations is not to be neglected, since emotion contagion for positive versus negative emotions has shown differential and opposing contributions to social well-being.^38^ In bipolar disorder risk, a differential perception and regulation of emotions depending on the emotional valence has been reported, including a retrospective bias in overestimating positive emotions and a heightened sensitivity to positively-valenced emotional distractors.^39^ In order to prevent potentially cancelling out unique and opposing associations depending on the emotion valence, we recommend to investigate both empathy for positive and negative emotions.

Our study findings on elevated mPFC and ACC activity in bipolar disorder risk, activated in situations demanding socio-cognitive functioning, serve as a relevant starting point for future investigations. Longitudinal research is indicated to follow up on the presumed adaptiveness of elevated mPFC and ACC activity. Individuals’ mental health development and neural activity should be jointly investigated to observe longitudinally whether these altered activations sustainably enable intact ToM or how they might be interrelated with potentially evolving socio-cognitive deficits. Finally, investigating the temporal stability of those aberrations – as they have similarly been demonstrated in longitudinal behavioural studies^18^ – would be crucial for testing the endophenotypic value of these neural variations. If these neural variations prove to reliably show in future investigations, in a next step, their malleability in response to mentalisation-based interventions could be studied.

## Supporting information

Choi et al. supplementary material Choi et al. supplementary material

## Data Availability

The data that support the findings of this study as well as the analytic code and research materials are available from the corresponding author on reasonable request.

## Supplementary material

The supplementary material is available online at https://doi.org/10.1192/bjp.2024.282.
