# Supplementary material for: Neural correlates of social affect and social cognition as risk markers of bipolar disorder
Source: Br J Psychiatry. 2025 Apr 30;228(2):126–32. doi: 10.1192/bjp.2024.282 (PMC12823450; doi:10.1192/bjp.2024.282)
Supplement: Choi et al. supplementary material [file S0007125024002824sup001.docx]

# Supplemental Information

## Study information and participant exclusion

Study data were acquired in the scope of a larger study (1,2), which was approved by the Ethics Committee of Dresden University (reference no. EK133042018). Besides the EmpaToM task and an online survey enquiring sociodemographic and psychometric data, further assessments not relevant to this preregistered study were conducted, including a laboratory-based spontaneous thought paradigm and a two-week ecological momentary assessment. The aim of the main study was to investigate narcissistic grandiosity and vulnerability in self and interpersonal aspects of functioning. Prospective participants were pre-screened with online questionnaires in order to maximize variance in narcissistic grandiosity and vulnerability. Considering that the common fluctuations between grandiose and vulnerable narcissism are associated with proneness to affective lability (3,4) and the latter being one of the most reliable prospective predictors of BD (5), this study sample is expected to exhibit valuable variance for present research objective. Previously published investigations based on this dataset (1,2) did not investigate this preregistered study’s variable of interest, that is, the Hypomanic Personality Scale (HPS; (6). For the present study, we analyzed experimental data on behavioral and neural measures of empathy and ToM and an online survey enquiring sociodemographic and psychometric data.

From the total number of study participants in the original study (*N* = 174), participant data were excluded in cases of incomplete study participation (*n* = 19), severe movement artifacts (*n* = 11), brain volumetric irregularities (*n* = 1), or current psychotropic medication and severe mental disorders, including schizophrenia and borderline personality disorder (*n* = 3), resulting in a final sample size of *N* = 140. Exclusion criteria were assessed based on self-reports in an initial online screening, and re-assessed by an experimenter using an in-house checklist for MRI studies upon arrival at the lab.”

## Psychometric properties of measures

### Hypomanic Personality Scale

For the psychometric assessment of hypomanic personality, the German Version of the Hypomanic Personality Scale (HPS) was administered (6,7). The 48-item self-report measure has good psychometric properties, with high internal consistency (Cronbach’s 𝛼 = .89) and 7-month test-retest reliability (*r*_tt_ = .77, *n* = 74; (6). The HPS has shown to be a valuable tool for detecting bipolar spectrum psychopathology as being both cross-sectionally and longitudinally (8–10) associated with BD. Considering the suggested dimensionality of hypomanic personality (11,12), both the HPS sum score as well as three subscale scores were calculated, representing social vitality (social potency and vivaciousness), mood volatility (negative, unpredictable mood states), and excitement (energetic and extremely cheerful mood; (12).

### Interpersonal Reactivity Index

For enquiring self-reported dispositional social affect and -cognition, the 16-item German version of the Interpersonal Reactivity Index (IRI; 8,9) was administered. Four subscales were calculated by the summation of respective item responses: perspective taking (tendency to adopt the psychological point of view of others), fantasy (tendency to transpose oneself imaginatively into the feelings and actions of fictitious characters), empathic concern (feeling of sympathy and concern for others), and personal distress (self-oriented feelings of personal anxiety and unease in tense interpersonal settings; 8). Good external validity and reliability (subscales’ Cronbach’s 𝛼 = .66 - .77) have been reported (14).

### Brief Symptom Inventory and life-time history of mental disorders

As a measure of symptom load, the German version of the Brief Symptom Inventory was analyzed (BSI; 10). For the present analyses, the global severity index (BSI-GSI) was considered, for which good internal consistency (Cronbach’s 𝛼 = .96) as well as convergent validity with established clinical rating scales have been reported (16). The presence of past or present mental disorders was additionally enquired via one item asking study participants whether they have ever been diagnosed with a mental disorder.

## ROI selection procedure

The multistep ROI selection procedure was administered as follows: First, from whole-brain analyses results on empathy and ToM in the largest EmpaToM dataset (17), those peak voxels with the largest cluster sizes (*k* > 50) or activation values (*Z* > 8.21; see 12) were selected.

In a second step, the number of peak voxels was further restricted by choosing those peak voxels showing an overlap in location with meta-analytic results on brain regions activated in naturalistic requirements of empathy and ToM (18). As in previous investigations (19), a significant overlap was defined as a Euclidean distance of less than 20 mm between the respective peak voxels. Resulting peak voxels were additionally required to have a minimum Euclidean distance of 20 mm to each other in order to ensure that separable ROIs were selected. Based on this procedure, three empathy- and three ToM-related ROIs were determined.

Lastly, we aimed to likewise consider findings reflecting neural aberrations in socio-affective and -cognitive tasks in specific association with BD risk. In light of the lack of reports on specific activation peaks regarding social affect and -cognition in BD risk, we considered findings from a systematic review reporting brain regions of neural aberrations of emotional and non-emotional cognition in BD-risk populations (20). Based on these anatomical overlaps, two additional ToM-ROIs were selected. An overview of all ROI can be found in Table S1.

**Table S1**

*Peak Coordinates of Empathy- and ToM-ROIs*

| AAL label | Anatomical description | MNI coordinates | | |
| --- | --- | --- | --- | --- |
|  |  | *x* | y | Z |
| Empathy-ROIs |  |  |  |  |
| Cerebelum_Crus2_L | L crus II of cerebellar hemisphere | -15 | -78 | -30 |
| Angular_L | L angular gyrus | -54 | -51 | 33 |
| Frontal_Inf_Orb_L | L inferior frontal gyrus pars orbitalis | -48 | 39 | -9 |
| Precuneus_L | L Precuneus | 0 | -63 | 36 |
| Angular_R | R angular gyrus | 63 | -48 | 33 |
| Frontal_Inf_Orb_R | R inferior frontal gyrus pars orbitalis | 51 | 30 | -6 |
|  |  |  |  |  |
| ToM-ROIs |  |  |  |  |
| Frontal_Inf_Tri_L | L inferior frontal gyrus, triangular part | -54 | 24 | 6 |
| Frontal_Sup_Medial_L | L superior frontal gyrus, medial | -9 | 54 | 24 |
| Cingulum_Post_L | L posterior cingulate gyrus | -6 | -51 | 30 |
| Temporal_Mid_L | L middle temporal gyrus | -51 | -57 | 24 |
| Temporal_Mid_L | L middle temporal gyrus | -48 | -30 | -3 |
| Cerebelum_Crus1_R | R crus I of cerebellar hemisphere | 27 | -78 | -33 |
| Frontal_Inf_Tri_R | R inferior frontal gyrus, triangular part | 54 | 30 | 3 |
| Frontal_Sub_Medial_R | R superior frontal gyrus, medial | 9 | 57 | 21 |

*Note.* If the coordinate did not match a brain region defined by the AAL, the closest brain region was computed. For ROI analyses, mean activation values from 7-mm spheres around the respective peak coordinates were extracted.

ToM = Theory of Mind, ROI = region of interest, AAL = Automated Anatomical Labelling, MNI = Montreal Neurologic Institute, L = left, R = right.

## fMRI data analysis

For the analysis of imaging data, we used SPM 12 (https://www.fil.ion.ucl.ac.uk/spm/software/spm12/). Preprocessing of fMRI data comprised slice-timing correction, realignment to the mean image, normalization based on coregistration with the T1-weighted image, spatial smoothing with a full-width-half-maximum Gaussian kernel of 8 mm, and the application of a high-pass filter of 128 s (see 16).

For the 1st level analysis, a General Linear Model (21) was fitted, including regressors for the video and question phases for each of the four conditions and for the rating phases, convolved with a hemodynamic response function (HRF). Six movement parameters were additionally added to the design matrix as covariates of no interest. For model estimation, the robust weighted least squares (RWLS) toolbox (22) was used.

In the 2nd level analysis, contrast images from the 1st level analysis were entered into one-sample t-tests for random effects analyses. Contrast images for the ‘Empathy contrast’ (negative vs. neutral videos) and the 'ToM contrast’ (ToM vs. nonToM questions) were then calculated by applying linear weights to the parameter estimates. For the exploratory whole-brain analyses, the HPS was added as a covariate into the model. In the scope of ROI analyses, activation values from 7-mm sphere ROIs were extracted with the marsbar toolbox (23).

**References**

1. Hildebrandt MK, Jauk E, Lehmann K, Maliske L, Kanske P. Brain activation during social cognition predicts everyday perspective-taking: A combined fMRI and ecological momentary assessment study of the social brain. NeuroImage. Februar 2021;227:117624.

2. Jauk E, Blum C, Hildebrandt M, Lehmann K, Maliske L, Kanske P. Psychological and neural correlates of social affect and cognition in narcissism: A multimethod study of self-reported traits, experiential states, and behavioral and brain indicators. Personality Disorders: Theory, Research, and Treatment [Internet]. 14. Dezember 2023 [zitiert 2. Januar 2024]; Verfügbar unter: http://doi.apa.org/getdoi.cfm?doi=10.1037/per0000645

3. Oltmanns JR, Widiger TA. Assessment of fluctuation between grandiose and vulnerable narcissism: Development and initial validation of the FLUX scales. Psychological Assessment. Dezember 2018;30(12):1612–24.

4. Pincus AL, Lukowitsky MR. Pathological Narcissism and Narcissistic Personality Disorder. Annu Rev Clin Psychol. 1. März 2010;6(1):421–46.

5. Taylor RH, Ulrichsen A, Young AH, Strawbridge R. Affective lability as a prospective predictor of subsequent bipolar disorder diagnosis: a systematic review. Int J Bipolar Disord. 1. November 2021;9(1):33.

6. Meyer TD, Drüke B, Hautzinger M. Hypomane Persönlichkeit - Psychometrische Evaluation und erste Ergebnisse zur Validität der deutschen Version der Chapman-Skala. Zeitschrift für Klinische Psychologie und Psychotherapie. 2000;(29):35–42.

7. Eckblad M, Chapman LJ. Development and Validation of a Scale for Hypomanic Personality. Journal of Abnormal Psychology. 1986;3(95):214–22.

8. Kwapil TR, Miller MB, Zinser MC, Chapman LJ, Chapman J, Eckblad M. A longitudinal study of high scorers on the Hypomanic Personality Scale. Journal of Abnormal Psychology. Mai 2000;109(2):222–6.

9. Walsh MA, Royal A, Brown LH, Barrantes-Vidal N, Kwapil TR. Looking for bipolar spectrum psychopathology: identification and expression in daily life. Comprehensive Psychiatry. Juli 2012;53(5):409–21.

10. Walsh MA, DeGeorge DP, Barrantes-Vidal N, Kwapil TR. A 3-year longitudinal study of risk for bipolar spectrum psychopathology. Journal of Abnormal Psychology. August 2015;124(3):486–97.

11. Schalet BD, Durbin CE, Revelle W. Multidimensional structure of the Hypomanic Personality Scale. Psychological Assessment. 2011;23(2):504–22.

12. Terrien S, Stefaniak N, Blondel M, Mouras H, Morvan Y, Besche-Richard C. Theory of mind and hypomanic traits in general population. Psychiatry Research. März 2014;215(3):694–9.

13. Davis MH. A Multidimensional Approach to Individual Differences in Empathy. 1980;

14. Paulus DC. DER SAARBRÜCKER PERSÖNLICHKEITSFRAGEBOGEN SPF (IRI) ZUR MESSUNG VON EMPATHIE. 2009;

15. Franke GH. Brief symptom inventory (BSI) von LR Derogatis:(Kurzform der SCL-90-R. 2000;

16. Geisheim C, Hahlweg K, Fiegenbaum W, Frank M, Schröder B, von Witzleben I. Das Brief Symptom Inventory (BSI) als Instrument zur Qualitätssicherung in der Psychotherapie. Diagnostica. 2002;(48):28–36.

17. Kanske P, Böckler A, Trautwein FM, Singer T. Dissecting the social brain: Introducing the EmpaToM to reveal distinct neural networks and brain–behavior relations for empathy and Theory of Mind. NeuroImage. November 2015;122:6–19.

18. Schurz M, Radua J, Tholen MG, Maliske L, Margulies DS, Mars RB, u. a. Toward a hierarchical model of social cognition: A neuroimaging meta-analysis and integrative review of empathy and theory of mind. Psychological Bulletin. März 2021;147(3):293–327.

19. Maliske LZ, Schurz M, Kanske P. Interactions within the social brain: Co-activation and connectivity among networks enabling empathy and Theory of Mind. Neuroscience & Biobehavioral Reviews. April 2023;147:105080.

20. Miskowiak KW, Kjærstad HL, Meluken I, Petersen JZ, Maciel BR, Köhler CA, u. a. The search for neuroimaging and cognitive endophenotypes: A critical systematic review of studies involving unaffected first-degree relatives of individuals with bipolar disorder. Neuroscience & Biobehavioral Reviews. Februar 2017;73:1–22.

21. Friston KJ, Holmes AP, Worsley KJ, Poline JP, Frith CD, Frackowiak RSJ. Statistical parametric maps in functional imaging: A general linear approach. Hum Brain Mapp. 1994;2(4):189–210.

22. Diedrichsen J, Shadmehr R. Detecting and adjusting for artifacts in fMRI time series data. NeuroImage. September 2005;27(3):624–34.

23. Brett M, Anton JL, Valabregue R, Poline JB. Region of interest analysis using an SPM toolbox. In Sendai, Japan: Available on CD-ROM in NeuroImage; 2002.

**Table S2**

*Results from whole-brain analysis on the effect of hypomanic personality traits on neural activity in the ToM contrast*

| set-level | |  | cluster-level | | | |  | peak-level | | | | |  | MNI coordinates | | |
| --- | --- | --- | --- | --- | --- | --- | --- | --- | --- | --- | --- | --- | --- | --- | --- | --- |
| p | c |  | p_FWE-corr_ | *q_FDR-corr_* | k*_E_* | *p_uncorr_* |  | p_FWE-corr_ | *q_FDR-corr_* | *T* | *(Z_E_)* | *p_uncorr_* |  | *x* | *y* | *Z* |
| .040 | 5 |  | .285 | .302 | 31 | .040 |  | .442 | .699 | 4.12 | 3.99 | .000 |  | 3 | 33 | 6 |
|  |  |  |  |  |  |  |  | .998 | .868 | 3.26 | 3.20 | .001 |  | 0 | 30 | -6 |
|  |  |  | .532 | .302 | 20 | .090 |  | .710 | .699 | 3.88 | 3.77 | .000 |  | -9 | -18 | -9 |
|  |  |  | .591 | .302 | 18 | .106 |  | .756 | .699 | 3.83 | 3.73 | .000 |  | -6 | 45 | 9 |
|  |  |  | .532 | .302 | 20 | .090 |  | .792 | .699 | 3.80 | 3.69 | .000 |  | -33 | 54 | 6 |
|  |  |  | .654 | .302 | 16 | .126 |  | .909 | .789 | 3.65 | 3.56 | .000 |  | 36 | 27 | 42 |
|  |  |  |  |  |  |  |  | .970 | .789 | 3.51 | 3.43 | .000 |  | 33 | 18 | 36 |
|  |  |  |  |  |  |  |  |  |  |  |  |  |  |  |  |  |

*Note.* The table shows three local maxima more than 8 mm apart.

ToM = Theory of Mind, MNI = Montreal Neurologic Institute, c = cluster, k_E_ = number of voxels per cluster.

**Table S3**

*Regression results using behavioral empathy as the criterion*

| Predictor | *b* | *b*  95% CI  [LL, UL] | *beta* | *beta*  95% CI  [LL, UL] | *sr^2^* | *sr^2^*  95% CI  [LL, UL] | *r* | Fit |
| --- | --- | --- | --- | --- | --- | --- | --- | --- |
| (Intercept) | 2.68** | [1.56, 3.81] |  |  |  |  |  |  |
| HPS | -0.01 | [-0.04, 0.02] | -0.06 | [-0.23, 0.11] | .00 | [-.02, .02] | -.06 |  |
| sex | -0.33* | [-0.62, -0.03] | -0.19 | [-0.36, -0.02] | .03 | [-.02, .09] | -.19* |  |
| age | 0.00 | [-0.02, 0.02] | 0.01 | [-0.16, 0.18] | .00 | [-.00, .00] | .05 |  |
|  |  |  |  |  |  |  |  | *R^2^*  = .040 |
|  |  |  |  |  |  |  |  | 95% CI[.00,.10] |
|  |  |  |  |  |  |  |  |  |

*Note.* A significant *b*-weight indicates the beta-weight and semi-partial correlation are also significant. *b* represents unstandardized regression weights. *beta* indicates the standardized regression weights. *sr^2^* represents the semi-partial correlation squared. *r* represents the zero-order correlation. *LL* and *UL* indicate the lower and upper limits of a confidence interval, respectively.
* indicates *p* < .05. ** indicates *p* < .01.

**Table S4**

*Results with from separate regression analyses with empathy-ROIs as the criterion*

| Criterion (AAL label) | Predictor | *b* | *b*  95% CI  [LL, UL] | *beta* | *beta*  95% CI  [LL, UL] | *sr^2^* | *sr^2^*  95% CI  [LL, UL] | *r* | Fit |
| --- | --- | --- | --- | --- | --- | --- | --- | --- | --- |
| *Frontal_Inf_Orb_L* | (Intercept) | 0.09 | [-0.37, 0.54] |  |  |  |  |  |  |
|  | HPS | 0.00 | [-0.01, 0.02] | 0.03 | [-0.14, 0.19] | .00 | [-.01, .01] | .03 |  |
|  | sex | 0.08 | [-0.04, 0.20] | 0.11 | [-0.06, 0.28] | .01 | [-.02, .05] | .13 |  |
|  | age | -0.00 | [-0.01, 0.00] | -0.08 | [-0.25, 0.09] | .01 | [-.02, .03] | -.11 |  |
|  |  |  |  |  |  |  |  |  | *R^2^*  = .024 |
|  |  |  |  |  |  |  |  |  | 95% CI[.00,.08] |
|  |  |  |  |  |  |  |  |  |  |
| *Frontal_Inf_Orb_R* | (Intercept) | 0.09 | [-0.40, 0.57] |  |  |  |  |  |  |
|  | HPS | -0.00 | [-0.02, 0.01] | -0.02 | [-0.19, 0.15] | .00 | [-.01, .01] | -.02 |  |
|  | sex | 0.06 | [-0.06, 0.19] | 0.08 | [-0.09, 0.26] | .01 | [-.02, .03] | .08 |  |
|  | age | 0.00 | [-0.01, 0.01] | 0.03 | [-0.14, 0.20] | .00 | [-.01, .01] | .02 |  |
|  |  |  |  |  |  |  |  |  | *R^2^*  = .007 |
|  |  |  |  |  |  |  |  |  | 95% CI[.00,.04] |
|  |  |  |  |  |  |  |  |  |  |
| Angular_L | (Intercept) | 0.11 | [-0.23, 0.45] |  |  |  |  |  |  |
|  | HPS | 0.00 | [-0.01, 0.01] | 0.08 | [-0.09, 0.25] | .01 | [-.02, .03] | .08 |  |
|  | sex | 0.02 | [-0.07, 0.11] | 0.05 | [-0.13, 0.22] | .00 | [-.01, .02] | .05 |  |
|  | age | -0.00 | [-0.01, 0.01] | -0.02 | [-0.19, 0.15] | .00 | [-.01, .01] | -.04 |  |
|  |  |  |  |  |  |  |  |  | *R^2^*  = .010 |
|  |  |  |  |  |  |  |  |  | 95% CI[.00,.04] |
|  |  |  |  |  |  |  |  |  |  |
| *Angular_R* | (Intercept) | 0.48* | [0.04, 0.91] |  |  |  |  |  |  |
|  | HPS | -0.01 | [-0.02, 0.01] | -0.07 | [-0.24, 0.10] | .01 | [-.02, .03] | -.07 |  |
|  | sex | -0.03 | [-0.14, 0.09] | -0.04 | [-0.21, 0.13] | .00 | [-.01, .01] | -.03 |  |
|  | age | -0.00 | [-0.01, 0.01] | -0.03 | [-0.20, 0.14] | .00 | [-.01, .01] | -.01 |  |
|  |  |  |  |  |  |  |  |  | *R^2^*  = .007 |
|  |  |  |  |  |  |  |  |  | 95% CI[.00,.03] |
|  |  |  |  |  |  |  |  |  |  |
| *Precuneus_L* | (Intercept) | -0.04 | [-0.62, 0.54] |  |  |  |  |  |  |
|  | HPS | 0.01 | [-0.01, 0.03] | 0.10 | [-0.07, 0.27] | .01 | [-.02, .04] | .10 |  |
|  | sex | 0.04 | [-0.11, 0.19] | 0.05 | [-0.13, 0.22] | .00 | [-.01, .02] | .04 |  |
|  | age | 0.00 | [-0.01, 0.01] | 0.03 | [-0.14, 0.20] | .00 | [-.01, .01] | .01 |  |
|  |  |  |  |  |  |  |  |  | *R^2^*  = .013 |
|  |  |  |  |  |  |  |  |  | 95% CI[.00,.05] |
|  |  |  |  |  |  |  |  |  |  |
| *Cerebelum_Crus2_L* | (Intercept) | 0.30 | [-0.06, 0.67] |  |  |  |  |  |  |
|  | HPS | -0.00 | [-0.02, 0.01] | -0.07 | [-0.24, 0.10] | .01 | [-.02, .03] | -.08 |  |
|  | sex | -0.03 | [-0.12, 0.07] | -0.05 | [-0.22, 0.12] | .00 | [-.01, .02] | -.06 |  |
|  | age | 0.00 | [-0.01, 0.01] | 0.02 | [-0.15, 0.19] | .00 | [-.01, .01] | .04 |  |
|  |  |  |  |  |  |  |  |  | *R^2^*  = .009 |
|  |  |  |  |  |  |  |  |  | 95% CI[.00,.04] |
|  |  |  |  |  |  |  |  |  |  |

*Note.* A significant *b*-weight indicates the beta-weight and semi-partial correlation are also significant. *b* represents unstandardized regression weights. *beta* indicates the standardized regression weights. *sr^2^* represents the semi-partial correlation squared. *r* represents the zero-order correlation. *LL* and *UL* indicate the lower and upper limits of a confidence interval, respectively.
ROI = region of interest, AAL = Automated Anatomical Labelling, MNI = Montreal Neurologic Institute, L = left, R = right.

* indicates *p* < .05. ** indicates *p* < .01.

**Table S5**

*Regression results using behavioral ToM as the criterion*

| Predictor | *b* | *b*  95% CI  [LL, UL] | *beta* | *beta*  95% CI  [LL, UL] | *sr^2^* | *sr^2^*  95% CI  [LL, UL] | *r* | Fit |
| --- | --- | --- | --- | --- | --- | --- | --- | --- |
| (Intercept) | 0.78** | [0.63, 0.94] |  |  |  |  |  |  |
| HPS | 0.00 | [-0.00, 0.01] | 0.05 | [-0.12, 0.21] | .00 | [-.01, .02] | .06 |  |
| sex | -0.05* | [-0.09, -0.01] | -0.20 | [-0.37, -0.04] | .04 | [-.02, .10] | -.18* |  |
| age | -0.00 | [-0.01, 0.00] | -0.13 | [-0.30, 0.04] | .02 | [-.02, .06] | -.10 |  |
|  |  |  |  |  |  |  |  | *R^2^*  = .052 |
|  |  |  |  |  |  |  |  | 95% CI[.00,.12] |
|  |  |  |  |  |  |  |  |  |

*Note.* A significant *b*-weight indicates the beta-weight and semi-partial correlation are also significant. *b* represents unstandardized regression weights. *beta* indicates the standardized regression weights. *sr^2^* represents the semi-partial correlation squared. *r* represents the zero-order correlation. *LL* and *UL* indicate the lower and upper limits of a confidence interval, respectively.

ToM = Theory of Mind.
* indicates *p* < .05. ** indicates *p* < .01.

**Table S6**

*Results with from separate regression analyses with ToM-ROIs as the criterion*

| Criterion (AAL label) | Predictor | *b* | *b*  95% CI  [LL, UL] | *beta* | *beta*  95% CI  [LL, UL] | *sr^2^* | *sr^2^*  95% CI  [LL, UL] | *r* | Fit |
| --- | --- | --- | --- | --- | --- | --- | --- | --- | --- |
| *Frontal_Inf_Tri_L* | (Intercept) | 0.58* | [0.02, 1.15] |  |  |  |  |  |  |
|  | HPS | -0.00 | [-0.02, 0.01] | -0.02 | [-0.19, 0.14] | .00 | [-.01, .01] | -.02 |  |
|  | sex | -0.12 | [-0.27, 0.02] | -0.14 | [-0.31, 0.03] | .02 | [-.03, .06] | -.13 |  |
|  | age | -0.00 | [-0.01, 0.01] | -0.05 | [-0.22, 0.12] | .00 | [-.01, .02] | -.02 |  |
|  |  |  |  |  |  |  |  |  | *R^2^*  = .020 |
|  |  |  |  |  |  |  |  |  | 95% CI[.00,.07] |
|  |  |  |  |  |  |  |  |  |  |
| *Cingulum_Post_L* | (Intercept) | 1.02** | [0.36, 1.68] |  |  |  |  |  |  |
|  | HPS | -0.01 | [-0.02, 0.01] | -0.05 | [-0.21, 0.12] | .00 | [-.01, .02] | -.02 |  |
|  | sex | 0.03 | [-0.14, 0.21] | 0.03 | [-0.14, 0.20] | .00 | [-.01, .01] | .07 |  |
|  | age | -0.02* | [-0.03, -0.00] | -0.20 | [-0.37, -0.03] | .04 | [-.02, .10] | -.20* |  |
|  |  |  |  |  |  |  |  |  | *R^2^*  = .044 |
|  |  |  |  |  |  |  |  |  | 95% CI[.00,.11] |
|  |  |  |  |  |  |  |  |  |  |
| *Temporal_Mid_L* | (Intercept) | 0.48 | [-0.06, 1.03] |  |  |  |  |  |  |
|  | HPS | 0.01 | [-0.01, 0.03] | 0.11 | [-0.06, 0.28] | .01 | [-.02, .05] | .12 |  |
|  | sex | -0.03 | [-0.18, 0.11] | -0.04 | [-0.21, 0.13] | .00 | [-.01, .01] | -.03 |  |
|  | age | -0.00 | [-0.02, 0.01] | -0.07 | [-0.25, 0.10] | .01 | [-.02, .03] | -.08 |  |
|  |  |  |  |  |  |  |  |  | *R^2^*  = .020 |
|  |  |  |  |  |  |  |  |  | 95% CI[.00,.07] |
|  |  |  |  |  |  |  |  |  |  |
| *Temporal_Mid_L* | (Intercept) | 0.32 | [-0.11, 0.74] |  |  |  |  |  |  |
|  | HPS | 0.00 | [-0.01, 0.02] | 0.06 | [-0.10, 0.23] | .00 | [-.02, .02] | .07 |  |
|  | sex | -0.11 | [-0.22, 0.00] | -0.17 | [-0.34, 0.00] | .03 | [-.03, .08] | -.16 |  |
|  | age | -0.00 | [-0.01, 0.01] | -0.03 | [-0.21, 0.14] | .00 | [-.01, .01] | -.01 |  |
|  |  |  |  |  |  |  |  |  | *R^2^*  = .032 |
|  |  |  |  |  |  |  |  |  | 95% CI[.00,.09] |
|  |  |  |  |  |  |  |  |  |  |
| *Cerebelum_Crus1_R* | (Intercept) | 0.83** | [0.23, 1.43] |  |  |  |  |  |  |
|  | HPS | -0.00 | [-0.02, 0.02] | -0.01 | [-0.18, 0.16] | .00 | [-.00, .00] | .00 |  |
|  | sex | -0.05 | [-0.21, 0.11] | -0.05 | [-0.23, 0.12] | .00 | [-.01, .02] | -.04 |  |
|  | age | -0.00 | [-0.02, 0.01] | -0.07 | [-0.24, 0.11] | .00 | [-.02, .03] | -.06 |  |
|  |  |  |  |  |  |  |  |  | *R^2^*  = .006 |
|  |  |  |  |  |  |  |  |  | 95% CI[.00,.03] |
|  |  |  |  |  |  |  |  |  |  |
| *Frontal_Inf_Tri_R* | (Intercept) | 0.31 | [-0.26, 0.87] |  |  |  |  |  |  |
|  | HPS | 0.01 | [-0.01, 0.02] | 0.07 | [-0.10, 0.23] | .00 | [-.02, .03] | .08 |  |
|  | sex | -0.08 | [-0.23, 0.07] | -0.09 | [-0.26, 0.08] | .01 | [-.02, .04] | -.07 |  |
|  | age | -0.01 | [-0.02, 0.00] | -0.10 | [-0.27, 0.08] | .01 | [-.02, .04] | -.09 |  |
|  |  |  |  |  |  |  |  |  | *R^2^*  = .020 |
|  |  |  |  |  |  |  |  |  | 95% CI[.00,.07] |
|  |  |  |  |  |  |  |  |  |  |
| *Frontal_Sup_Medial_L* | (Intercept) | 0.28 | [-0.22, 0.78] |  |  |  |  |  |  |
|  | HPS | 0.01 | [-0.00, 0.02] | 0.12 | [-0.05, 0.29] | .01 | [-.02, .05] | .12 |  |
|  | sex | -0.01 | [-0.14, 0.12] | -0.01 | [-0.18, 0.16] | .00 | [-.00, .00] | -.00 |  |
|  | age | -0.00 | [-0.01, 0.01] | -0.04 | [-0.22, 0.13] | .00 | [-.01, .02] | -.06 |  |
|  |  |  |  |  |  |  |  |  | *R^2^*  = .017 |
|  |  |  |  |  |  |  |  |  | 95% CI[.00,.06] |
|  |  |  |  |  |  |  |  |  |  |
| *Frontal_Sub_Medial_R* | (Intercept) | 0.16 | [-0.34, 0.66] |  |  |  |  |  |  |
|  | HPS | 0.01* | [0.00, 0.03] | 0.17 | [0.00, 0.34] | .03 | [-.03, .08] | .18* |  |
|  | sex | 0.01 | [-0.12, 0.15] | 0.02 | [-0.15, 0.19] | .00 | [-.01, .01] | .03 |  |
|  | age | -0.00 | [-0.01, 0.01] | -0.06 | [-0.23, 0.11] | .00 | [-.02, .02] | -.09 |  |
|  |  |  |  |  |  |  |  |  | *R^2^*  = .036 |
|  |  |  |  |  |  |  |  |  | 95% CI[.00,.10] |
|  |  |  |  |  |  |  |  |  |  |
|  |  |  |  |  |  |  |  |  |  |

*Note.* A significant *b*-weight indicates the beta-weight and semi-partial correlation are also significant. *b* represents unstandardized regression weights. *beta* indicates the standardized regression weights. *sr^2^* represents the semi-partial correlation squared. *r* represents the zero-order correlation. *LL* and *UL* indicate the lower and upper limits of a confidence interval, respectively.
ToM = Theory of Mind, ROI = region of interest, AAL = Automated Anatomical Labelling, MNI = Montreal Neurologic Institute, L = left, R = right.

* indicates *p* < .05. ** indicates *p* < .01.
